# Complete genome sequence data of *Xylella fastidiosa* subspecies *multiplex* ST88 and ST89 indicate distinct introductions in France

**DOI:** 10.1016/j.dib.2025.111816

**Published:** 2025-06-21

**Authors:** Amandine Cunty, Jessica Dittmer, Déborah Merda, Bruno Legendre, Benoit Remenant, Yannick Blanchard, Sophie Cesbron, Marie-Agnès Jacques, Pascal Gentit, Anne-Laure Boutigny

**Affiliations:** a ANSES, Plant Health Laboratory, BVO Unit, Angers, France; b UMR 1345, University of Angers, Institut Agro, INRAE, IRHS, SFR Quasav, Angers, France; c ANSES, Food Safety Laboratory, SPAAD Unit, Maisons-Alfort, France; d ANSES, Ploufragan Laboratory, VGB Unit, Ploufragan, France

**Keywords:** Sequence type, Quarantine bacteria, Long and short reads, PacBio, Illumina

## Abstract

*Xylella fastidiosa* is a Gram-negative bacterium native to the Americas and classified as a priority pest under EU regulations. This xylem-limited plant pathogenic bacterium has a wide host range and is transmitted by insect vectors. Since 2013, *X. fastidiosa* has been identified in several European countries including Italy, France, Spain and Portugal, with different subspecies and sequence types (ST) detected. Since 2015, most strains identified in France are of the subspecies *multiplex,* specifically ST6 and ST7. Two new STs of *X. fastidiosa* subsp. *multiplex,* ST88 and ST89, were recently detected in the region Provence-Alpes-Côte d’Azur (PACA), and one strain of each ST has been isolated from infected plants. To investigate the phylogenetic relationships between the four STs present in France, a complete circular genome and a single-contig genome were assembled for the ST89 and ST88 strains, respectively, by combining PacBio and Illumina sequencing data. A phylogenomic analysis was performed to investigate the phylogenetic position and potential origin of these new strains. This data article contributes to improve our knowledge of the diversity and origin of *X. fastidiosa* subsp. *multiplex* in France and Europe.

Specifications Table

**Table:** 

Subject	Microbiology
Specific subject area	Bacteriology, Fastidious bacteria, Genomics
Type of data	Table, Figure Genomic data, Raw, Analyzed, Filtered, Processed
Data collection	Genomic DNA extraction: Wizard Genomic DNA Purification Kit (Promega) and QuickPick^TM^ SML Plant DNA kit (Bio-Nobile) Genome sequencing platforms: Illumina MiSeq and PacBio Bioinformatic tools: Canu v2.1.1, Flye v2.8.1, Circlator v1.5.6, Polca from the MaSuRCa toolkit v4.0.9, CheckM v1.1.6, Bakta v1.5.1
Data source location	Plant Health Laboratory of ANSES, Angers, France IRHS, Angers, France
Data accessibility	Repository name: NCBI (www.ncbi.nlm.nih.gov) Data identification number: Bioprojects PRJNA1234384 and PRJNA1234386 Direct URL to data: LSV 52.37: https://www.ncbi.nlm.nih.gov/bioproject/PRJNA1234384 https://www.ncbi.nlm.nih.gov/biosample/SAMN47296926 https://www.ncbi.nlm.nih.gov/sra/SRX27952896 https://www.ncbi.nlm.nih.gov/sra/SRX27952897 https://www.ncbi.nlm.nih.gov/sra/SRX27952898 LSV 52.52: https://www.ncbi.nlm.nih.gov/bioproject/PRJNA1234386 https://www.ncbi.nlm.nih.gov/biosample/SAMN47297024 https://www.ncbi.nlm.nih.gov/sra/SRX27953175 https://www.ncbi.nlm.nih.gov/sra/SRX27953176 https://www.ncbi.nlm.nih.gov/sra/SRX27953177 Instructions for accessing these data: The complete genome sequences of *Xylella fastidiosa* subsp. *multiplex* strains LSV52.37 (ST88) and LSV52.52 (ST89) are available in the National Centre for Biotechnology Information (NCBI) database (Bioproject PRJNA1234384, Biosample SAMN47296926 for LSV52.37 and Bioproject PRJNA1234386, Biosample SAMN47297024 for LSV52.52). The raw data are available under the following accession numbers: SRR32648475 to SRR32648477 for LSV52.37 and SRR32648754 to SRR32648756 for LSV52.52.
Related research article	A. Cunty, B. Legendre, P. de Jerphanion, C. Dousset, A. Forveille, S. Paillard, V. Olivier, Update of the *Xylella fastidiosa* outbreak in France: two new variants detected and a new region affected, Eur. J. Plant Pathol. 163 (2022) 505–510. 10.1007/s10658-022-02492-z.

## 1 Value of the Data

• Data report on the high-quality genome sequences of two strains of *X. fastidiosa* subsp. *multiplex* ST88 and ST89 isolated in France.
• The data extend genomic data available for European strains of *X. fastidiosa* subsp. *multiplex* and are valuable for comparative genomics.
• The data provide a better overview of the diversity and origin of *X. fastidiosa* subsp. *multiplex* in France and Europe.
• The data support the hypothesis of two distinct introductions from the USA to France for both ST88 and ST89, rather than both ST88 and ST89 having evolved from strains already present in France. These results are in accordance with the MLST analysis previously performed on these two strains [1].

## 2 Background

*Xylella fastidiosa* is a Gram-negative, xylem-limited phytopathogenic bacterium with a broad host range, which causes significant epidemics and economic losses in the Americas and Europe [2]. Despite being native to the Americas [3], since 2013 *X. fastidiosa* has been identified in various plant species in European countries with a Mediterranean climate, such as Italy, France, Spain and Portugal, with different subspecies and sequence types (ST) detected [2,4]. These detections are the result of multiple independent introductions likely originating from the USA [4,5]. In France, the subspecies *multiplex* was first detected in 2015 in Corsica and in the region Provence-Alpes-Côte d’Azur (PACA) (ST6 and ST7) and in 2020 in the Occitanie region (ST6) [1,6]. Also in 2020, two new STs of *X. fastidiosa* subsp. *multiplex* were identified in the PACA region at two different locations and in different host plants: (i) ST88 was detected on *Polygala myrtifolia, Hebe sp., Osteospermum ecklonis, Lavandula x intermedia, Coronilla glauca* and *Euryops chrysanthemoides* and (ii) ST89 was detected on *Myoporum sp.* and *Viburnum tinus* [1]. Here, we report the high-quality genome sequence of an ST88 strain and the complete circular genome sequence of an ST89 strain by combining PacBio and Illumina sequencing data. In addition, we performed a phylogenomic analysis of the subspecies *multiplex* to investigate the phylogenetic position and potential origin of these new strains.

## 3 Data Description

**Fig. 1:**
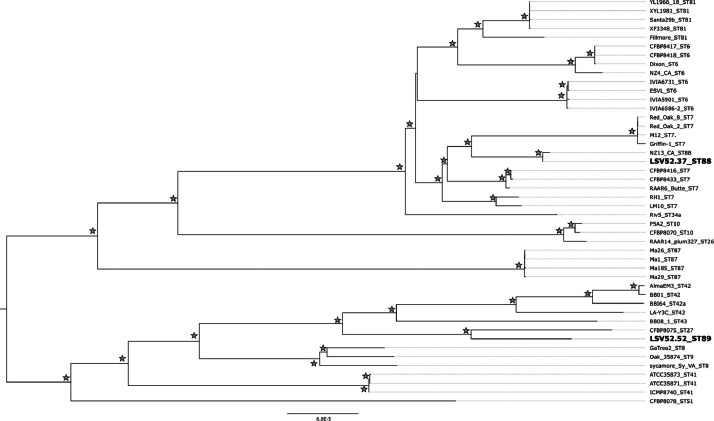
Phylogenomic tree showing the relationships of *X. fastidiosa* subsp. *multiplex* strains using maximum likelihood analysis. The tree was visualized using FigTree. Strains sequenced in this study are in bold. Bootstraps > 80% are represented by grey stars.

Long- and short-reads were obtained *via* the PacBio HiFi and Illumina platforms, respectively. The final assemblies resulted in a single non-circularized contig for the ST88 strain LSV52.37 and a complete circular genome for the ST89 strain LSV52.52 (Table 1). No plasmids were detected in either genome. The genome of the ST88 strain was longer than the genome of the ST89 strain (2.69 Mbp and 2.52 Mbp, respectively) but both genomes are in the typical size range for *X. fastidiosa* genomes. The number of pseudogenes was very low (25 and 32, respectively) and CheckM confirmed a high genome completeness and absence of contamination (Table 1). The genome assemblies and the raw sequencing data were deposited in the NCBI databases under BioProject numbers PRJNA1234384 for *X. fastidiosa* subsp. *multiplex* ST88 (strain LSV52.37) and PRJNA1234386 for *X. fastidiosa* subsp. *multiplex* ST89 (strain LSV 52.52). A phylogenomic analysis based on SNPs was performed using the two genomes obtained in this study and 44 genome sequences representing the genetic diversity of the subspecies *multiplex* available in GenBank as of 12/12/2024 (Table 2, Fig. 1).

**Table 1:** Genome assembly and annotation metrics of the two *Xylella fastidiosa* subsp. *multiplex* ST88 and ST89 strains.

Metrics	LSV52.37 (ST88)	LSV52.52 (ST89)
Nb of reads (PacBio/Illumina)	56,996 / 3,967,328	69,832 / 3,895,010
Genome size (bp)	2,698,430	2,524,893
Nb of contigs	1	1
GC (%)	51.77	51.81
Coverage (long reads)	171	243
Coverage (short reads)	282	256
CDS	2679	2409
Pseudogenes	25	32
rRNA	6	6
tRNA	55	54
tmRNA	1	1
ncRNA	9	6
Regulatory ncRNA	2	2
CheckM Completeness (%)	99.28	99.64
CheckM Contamination (%)	0.00	0.00
BioProject	PRJNA1234384	PRJNA1234386

**Table 2:** Genome sequences of *X. fastidiosa* subsp. *multiplex* strains available in GenBank used for the phylogenomic analysis.

Strain name	ST	Host plant	Location	Accession
CFBP8418	6	*Spartium junceum*	France: Corsica	GCF_042244175.1
CFBP8417	6	*Spartium junceum*	France: Corsica	GCF_042244685.1
Dixon	6	*Prunus dulcis*	USA: California	GCF_029626005.1
ESVL	6	*Prunus dulcis*	Spain: Alicante	GCF_004023385.1
IVIA5901	6	*Prunus dulcis*	Spain: Alicante	GCF_004023395.2
IVIA6586-2	6	*Helichrysum italicum*	Spain: Alicante	GCF_009669335.1
IVIA6731	6	*Helichrysum italicum*	Spain: Alicante	GCF_009669375.1
NZ4_CA	6	*Brachyglottis compacta*	USA: California	GCF_043355825.1
CFBP8416	7	*Polygala myrtifolia*	France: Corsica	GCF_001971475.1
CFBP8433	7	*Cistus monspeliensis*	France: Corsica	GCF_028752655.1
Griffin-1	7	*Quercus rubra*	USA: Georgia	GCA_000466025.1
LM10	7	*Olea europaea*	USA: California	GCF_012974145.1
M12	7	*Prunus dulcis*	USA: California	GCF_000019325.1
RAAR6 Butte	7	*Prunus dulcis*	USA: California	GCF_009695485.1
Red Oak 2	7	*Quercus rubra*	USA: Georgia	GCF_015475935.1
Red Oak 8	7	*Quercus rubra*	USA: Georgia	GCF_021459885.1
RH1	7	*Olea europaea*	USA: California	GCF_012974125.1
sycamore Sy-VA	8	*Platanus occidentalis*	USA: Virginia	GCF_000732705.1
GaTree2	8	*Carya illinoinensis*	USA: Georgia	GCA_042862445.1
Oak 35874	9	*Quercus sp.*	USA: Washington	GCF_021459905.1
CFBP8070	10	*Prunus sp.*	USA: Georgia	GCF_042243345.1
P5A2	10	*Prunus persica*	USA: Alabama	GCA_022548865.1
RAAR14 plum327	26	*Prunus domestica*	Brazil: Rio Grande do Sul	GCF_009695495.1
CFBP8075	27	*Prunus sp.*	USA: California	GCF_042242545.1
Riv5	34	*Prunus cerasifera*	USA: California	GCF_015475955.1
CFBP8173 (ATCC 35871)	41	*Prunus salicina*	USA: Georgia	GCF_042241145.1
ICMP8740	41	*Platanus occidentalis*	USA: Washington	GCF_028735895.1
CFBP8068 (ATCC 35873)	41	*Ulmus sp.*	USA: Washington	GCF_042241895.1
AlmaEm3	42	*Vaccinium sp.*	USA: Georgia	GCF_018069645.1
BB01	42	*Vaccinium corymbosum*	USA: Georgia	GCF_001886315.1
LA-Y3C	42	*Vaccinium virgatum*	USA: Louisiana	GCF_021459845.1
BBI64	42	*Vaccinium sp.*	USA: Georgia	GCA_006369955.1
BB08-1	43	*Vaccinium ``Windsor''*	USA: Florida	GCF_018069665.1
CFBP8078	51	*Vinca sp.*	USA: Florida	GCF_004016365.1
Fillmore	81	*Olea europaea*	USA: California	GCF_012974105.1
XF3348	81	*Prunus dulcis*	Spain: Majorca	GCF_042239545.1
XYL1966/18	81	*Olea europaea*	Spain: Minorca	GCF_042238405.1
XYL1981	81	*Ficus carica*	Spain: Majorca	GCF_009669455.1
Santa29b	81	*Santolina chamaecyparissus*	Spain: Minorca	GCF_042240315.1
Ma1	87	*Rhamnus alaternus*	Italy: Tuscany	GCF_018449155.1
Ma26	87	*Spartium junceum*	Italy: Tuscany	GCF_018449175.1
Ma29	87	*Prunus dulcis*	Italy: Tuscany	GCF_018449135.1
Ma185	87	*Polygala myrtifolia*	Italy: Tuscany	GCF_018449105.1
NZ13_CA	88	*Pomaderris prunifolia*	USA: California	GCF_043195255.1

This analysis revealed that the ST88 strain (LSV52.37) isolated from *Polygala myrtifolia* in France clustered with another ST88 strain (NZ13_CA) recently isolated from *Pomaderris prunifolia* in California (USA) [7]. The two ST88 strains formed a highly-supported clade most closely related to ST7 strains from Red Oak and almond isolated in the USA. In contrast, the ST89 strain (LSV52.52) was closely-related to American ST27 infecting almond and plum.

## 4 Experimental Design, Materials and Methods

### 4.1 Bacterial Culture

*Xylella fastidiosa* subsp. *multiplex* strain LSV52.37 (ST88) was isolated in 2020 from *Polygala myrtifolia* in Saint-Raphaël (PACA region) and LSV52.52 (ST89) was isolated in 2021 from *Myoporum sp.* in Villeneuve-Loubet (PACA region) on modified PWG (Periwinkle Wilt-Gelrite) medium [2]. They are stored at -80°C in the laboratory collection of the ANSES Plant Health Laboratory.

### 4.2 DNA Extraction and Library Preparation

For PacBio long-read sequencing, bacteria were grown on solid PWG medium at 28°C for seven days. The cultures were replated twice to obtain sufficient bacterial cells. Bacterial cells from these plate cultures were suspended in 10 mL of sterile water with DO_600_ 0.3-0.6. Genomic DNA was extracted from these bacterial suspensions using the Wizard Genomic DNA Purification Kit (Promega, Madison, USA), following the protocol for Gram-positive bacteria with some modifications to improve cell lysis and DNA purity: the initial bacterial cell pellet was washed once in 2 mL of sterile water before being resuspended in 480 µL EDTA (50 mM, pH=8). Lysozyme (120 µL, 10 mg/mL) was then added, followed by incubation at 37°C for 60 min. 35 µL of Pronase (5 mg/mL) was added and the samples incubated overnight at 37°C. Next, 150 µL of 10% SDS (w/v) was added and the samples were incubated at 37°C for 45 min. Nuclei Lysis Solution (800 µL) was then added and the samples were incubated at 80°C for 5 min. Following RNase A treatment at 37°C for 60 min, 270 µL of Protein Lysis Solution was added and incubated on ice for 20 min. After centrifugation at 16,000 g for 6 min, DNA was precipitated in 1 volume of isopropanol, the DNA pellet was washed once with 70% ethanol (v/v) and resuspended in DNA Rehydration Solution. DNA quality was verified using a Nanodrop One (Thermo Fisher Scientific, Waltham, USA) and DNA concentration was quantified using the Qubit dsDNA Broad Range assay kit (Invitrogen, Waltham, USA). Library preparation and sequencing on the PacBio HiFi platform were performed by the Gentyane sequencing facility at Clermont-Ferrand, France. For Illumina sequencing, bacteria were grown on solid modified PWG medium at 28°C for seven days. The cultures were replated twice to obtain sufficient bacterial cells. Bacterial cells from these plate cultures were suspended in 1 mL of sterile water at a concentration of 10^9^ CFU/mL. Genomic DNA was extracted from these bacterial suspensions using the QuickPick^TM^ SML Plant DNA kit (Bio-Nobile, Pargas, Finland). Illumina sequencing libraries were generated as previously described [8].

### 4.3 Sequencing Analysis

Long-read assemblies were performed using both Canu v2.1.1 [9] and Flye v2.8.1 [10]. For the strain LSV52.37 (ST88), the most contiguous assembly was obtained using Canu with the parameters *genomeSize=2.7m, minReadLength=2000, minOverlapLength=1000*, producing two contigs. Using the Illumina reads, these two contigs were scaffolded into a single contig with Redundans v0.14 [11] with the parameters *–noreduction –limit 1*. Circularization was attempted using Circlator v1.5.6 [12] but was not successful. The genome of the strain LSV52.52 (ST89) was directly assembled into a single circular contig using Flye with the parameters *–pacbio-hifi, -g 2.*7*m* and –*min-overlap 1000*. The genome was rotated to start with the gene *dnaA* using Circlator’s *fixstart* function. Both assemblies were polished with the Illumina reads using Polca from the MaSuRCa toolkit v4.0.9 [13], but no errors were found, confirming the high quality of the PacBio HiFi assemblies. Genome completeness and contamination was verified using CheckM v1.1.6 [14] and both genomes were annotated using Bakta v1.5.1 [15] (Table 1). Coverage was estimated using Mosdepth [16] as implemented on Galaxy (usegalaxy.org). Default parameters were used for all bioinformatics tools unless stated otherwise.

### 4.4 Phylogenomic Analysis

Multiple genome alignment of all assemblies (Table 2) was performed using Parsnp v1.5.6 [17]. An in-house python script (supplementary data) was used to concatenate blocks in the xmfa file to obtain a fasta file. Recombination tracts were removed from the alignment using ClonalFrameML v1.12 [18]. The maximum likelihood phylogenetic tree was obtained using IQ-TREE v2.0.3 [19]. The best substitution model was inferred directly by JModelTest [20] implemented in IQ-TREE, and the values of branch support were obtained by bootstrap method using 1000 replicates. The final phylogenetic tree was visualized using FigTree v1.4.4 (https://github.com/rambaut/figtree).

## Limitations

None.

## Ethics Statement

The authors have read and follow the *ethical requirements* for publication in Data in Brief. The current work does not involve human subjects, animal experiments, or any data collected from social media platforms

## CRediT authorship contribution statement

**Amandine Cunty:** Conceptualization, Methodology, Writing – original draft. **Jessica Dittmer:** Methodology, Writing – original draft. **Déborah Merda:** Formal analysis, Writing – review & editing. **Bruno Legendre:** Writing – review & editing. **Benoit Remenant:** Writing – review & editing. **Yannick Blanchard:** Writing – review & editing. **Sophie Cesbron:** Methodology, Writing – review & editing. **Marie-Agnès Jacques:** Funding acquisition, Writing – review & editing. **Pascal Gentit:** Funding acquisition, Writing – review & editing. **Anne-Laure Boutigny:** Conceptualization, Methodology, Writing – review & editing.

## Data Availability

NCBIXylella fastidiosa ST89 genome sequencing and assembly (Original data). NCBIXylella fastidiosa ST88 genome sequencing and assembly (Original data).

## Appendix Supplementary materials

Image, application 1
